# Metastasis of ovarian cancer to the bile duct: a case report

**DOI:** 10.1186/s40792-019-0659-9

**Published:** 2019-06-20

**Authors:** Masahiro Shijo, Koji Fukase, Hideo Ohtsuka, Kyohei Ariake, Kunihiro Masuda, Masaharu Ishida, Masamichi Mizuma, Kei Nakagawa, Hiroki Hayashi, Takanori Morikawa, Fuyuhiko Motoi, Takeshi Naitoh, Michiaki Unno

**Affiliations:** Department of Surgery, Tohoku University Graduate School of Medicine, 1-1 Seiryo-machi, Aoba-ku, Sendai, Miyagi 980-8574 Japan

**Keywords:** Ovarian cancer, Bile duct metastasis, Intrabiliary metastasis, Hilar cholangiocarcinoma

## Abstract

**Background:**

Ovarian cancer typically spreads along the peritoneum or metastasizes through the blood or lymphatic stream. The bile duct is an extremely rare site of ovarian cancer-associated metastases.

**Case presentation:**

A 55-year-old female underwent surgery for advanced left ovarian cancer 2 years ago. She was diagnosed with ovarian serous adenocarcinoma with multiple peritoneal metastases. She received chemotherapy for the residual peritoneal metastases. She achieved a clinical complete response and was followed up with imaging examinations for 1 year. She then complained of dark urine, yellowish discoloration of the eyes, and weight loss. Computed tomography showed an approximately 10-mm solid tumor at the hepatic hilum. Simultaneously, multiple peritoneal metastases were detected in the abdominal and pelvic cavity. Intraductal ultrasonography suggested that the hepatic hilum tumor was located in the bile duct wall. Tumor biopsy and brush cytology of the bile duct indicated atypical cells suspicious for carcinoma. After percutaneous transhepatic portal embolization, she underwent right hepatectomy and extrahepatic bile duct resection for the hepato-hilar tumor. The histopathological features were dysplastic cells with hyperchromatic nuclei and no dysplastic cells in the native biliary epithelium. Immunohistochemical staining revealed that the tumor cells were positive for CK-7 and WT-1 and negative for CK-20 and ER. These results suggested that the tumor was a metastasis of the ovarian serous adenocarcinoma.

**Conclusion:**

This may be the first case of ovarian cancer metastasis to the bile duct. While it is extremely rare, ovarian cancer may metastasize to the hepatic duct, mimicking hilar cholangiocarcinoma.

## Background

Epithelial ovarian cancer is the fourth most common cause of cancer death among females in developed countries [1]. Though treatments with surgery and chemotherapy such as paclitaxel and carboplatin have developed, improvements in the 5-year overall survival rate have not been remarkable in the last two decades. About 60–80% of women with advanced ovarian cancer will have tumor progression or a recurrence [2]. Ovarian cancer typically spreads along the peritoneum or metastasizes through the blood or lymphatic stream. Common sites of metastasis include the peritoneum, abdominal lymph nodes, bowel, liver, and lung [3]. There are some reports that metastatic lymph nodes on the hepatic hilum invaded the extrahepatic bile duct and caused jaundice in patients with ovarian cancer [4] [5]. Metastasis to the bile duct from ovarian cancer is extremely rare. To our knowledge, this may be the first report of metastasis to the bile duct from primary ovarian cancer.

## Case presentation

A 55-year-old female underwent total hysterectomy, bilateral salpingo-oophorectomy, omentectomy, and partial transverse colectomy for advanced left ovarian cancer 2 years ago. She was diagnosed with ovarian serous adenocarcinoma and multiple peritoneal metastases based on operative and pathological findings (International Federation of Gynecology and Obstetrics: FIGO stage IIIc). After the operation, she received chemotherapy with paclitaxel and carboplatin, which is regarded as the standard therapy for advanced ovarian cancer, against the residual peritoneal metastases for 8 months. After the chemotherapy, she achieved a clinical complete response on abdominal computed tomography (CT). In addition, the serum tumor marker cancer antigen 125 (CA 125) level, which was elevated to 274.3 U/ml before the treatment, decreased to the normal range.

**Fig. 1:**
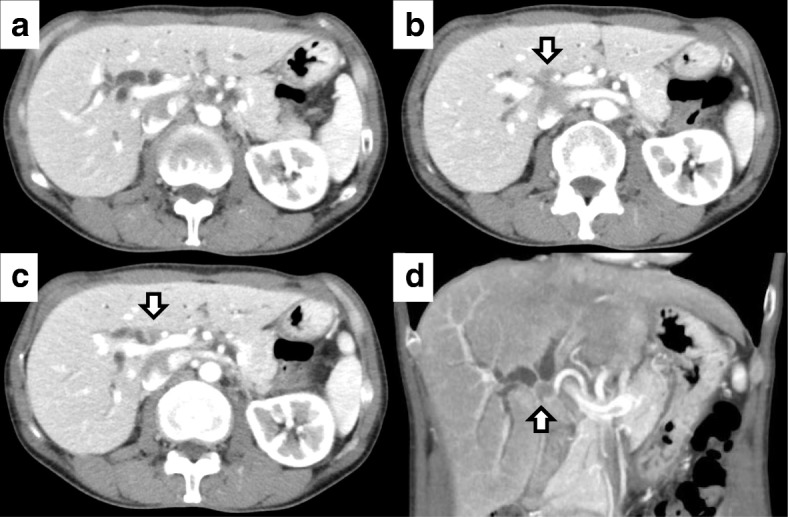
Contrast-enhanced abdominal computed tomography (CT) findings. Contrast-enhanced CT showed an approximately 10-mm high-attenuation intraductal mass at the hepatic hilum (arrow) and dilation of the intrahepatic bile duct. **a**–**c** Axial views. **d** Coronal view

**Fig. 2:**
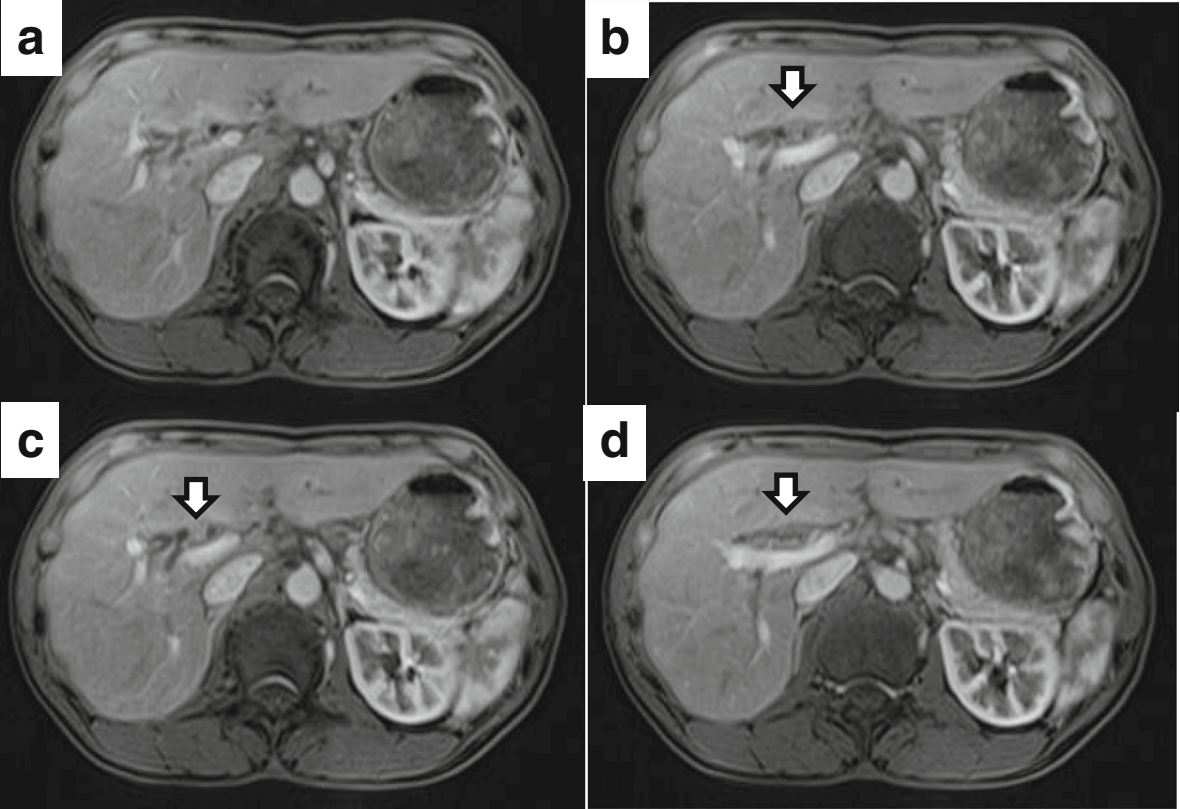
Contrast-enhanced magnetic resonance imaging (MRI) findings. **a**–**d** Contrast-enhanced MRI showed dilation of the intrahepatic bile duct and an approximately 10-mm high-attenuation intraductal mass at the hepatic hilum (arrow) and dilation of the intrahepatic bile duct

**Fig. 3:**
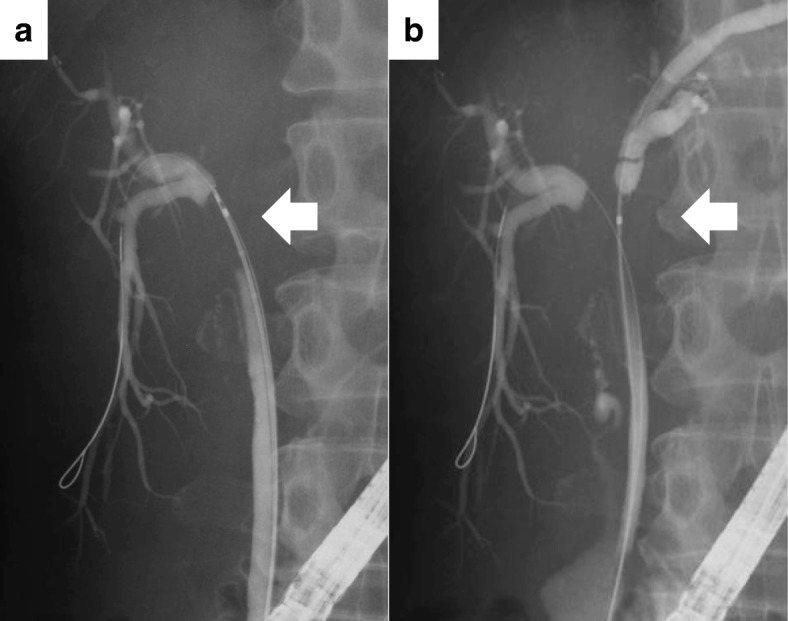
Endoscopic retrograde cholangiography (ERC) findings. **a**, **b** ERC revealed the defect at the bifurcation of the common hepatic duct (Bismuth-Corlette classification type II)

**Fig. 4:**
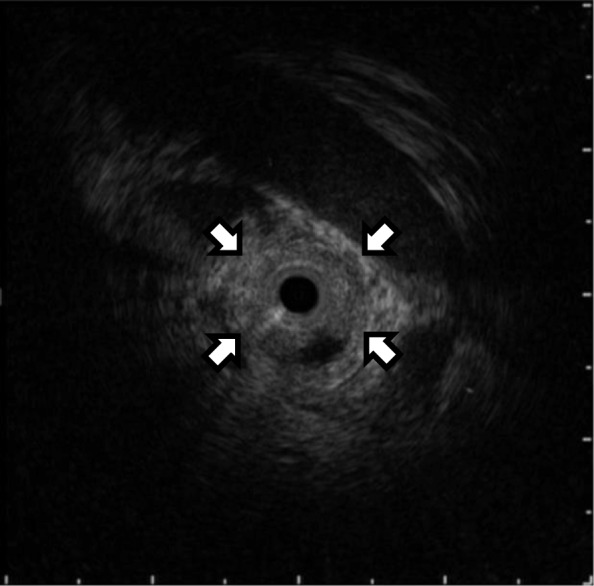
Intraductal ultrasonography (IDUS) findings. Intraductal ultrasonography (IDUS) suggested the tumor (arrow) was located in the bile duct wall

Two years after the initial surgical treatment for the ovarian cancer, she complained of dark urine, yellowish discoloration of the eyes, and loss of body weight. The serum CA125 level increased to 50.9 U/ml. Carbohydrate antigen (CA) 19–9 and carcinoembryonic antigen (CEA) levels were normal. The total bilirubin level was elevated at 10.4 mg/dl. Abdominal CT showed intrahepatic bile duct dilatation due to an approximately 10-mm solid tumor at the hepatic hilum (Fig. 1). Simultaneously, several irregular peritoneal nodules (less than 15 mm in size) were detected in the pelvic cavity. Fluorodeoxyglucose-positron emission tomography (FDG-PET) showed uptake in the hepatic hilum tumor (standardized uptake value (SUV) max 8.2) and the pelvic nodules (SUV max 4.7). Contrast-enhanced magnetic resonance imaging (MRI) findings also showed a high-attenuation intraductal mass at the hepatic hilum and dilation of the intrahepatic bile duct (Fig. 2). Endoscopic retrograde cholangiography (ERC) revealed the defect at the bifurcation of the common hepatic duct (Bismuth-Corlette classification type II) and endoscopic nasobiliary drainage was performed for obstructive jaundice (Fig. 3). Intraductal ultrasonography (IDUS) suggested the tumor was located in the bile duct wall (Fig. 4). Tumor biopsy and brush cytology of the bile duct indicated atypical cells suspicious for carcinoma; however, a definite histopathological diagnosis could not be obtained because of the limited biopsy samples. Preoperatively, she was diagnosed with a hepato-hilar bile duct tumor, concurrent with recurrent peritoneal metastasis of ovarian cancer. The recurrent peritoneal dissemination was regarded to be controllable by paclitaxel and carboplatin, because it developed 14 months after stopping chemotherapy. Curative resection was suggested for treatment of the hepato-hilar bile duct tumor, because a definite histopathological diagnosis was not obtained and primary cholangiocarcinoma could not be ruled out. The abdominal CT demonstrated that the tumor was in contact with the right hepatic artery, suggesting arterial infiltration. We planned a right hepatectomy and performed preoperative percutaneous transhepatic portal embolization (PTPE). The CT volumetry 3 weeks after the PTPE demonstrated that the volume of the left lobe increased from 390 to 511 ml. She underwent right hepatectomy with caudate lobectomy and extrahepatic bile duct resection.

**Fig. 5:**
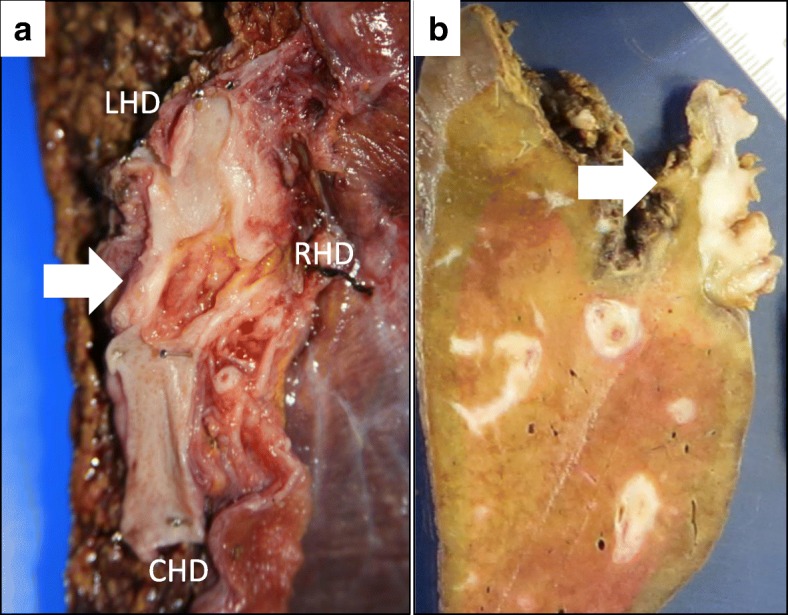
Macroscopic findings. A 33 × 15-mm well-demarcated tumor occupied the bile duct at the hepatic hilum (arrow). **a** Gross examination of the resected specimen. **b** Cut surface of the hepatic hilum. *CHD* common hepatic duct. *RHD* right hepatic duct. *LHD* left hepatic duct

**Fig. 6:**
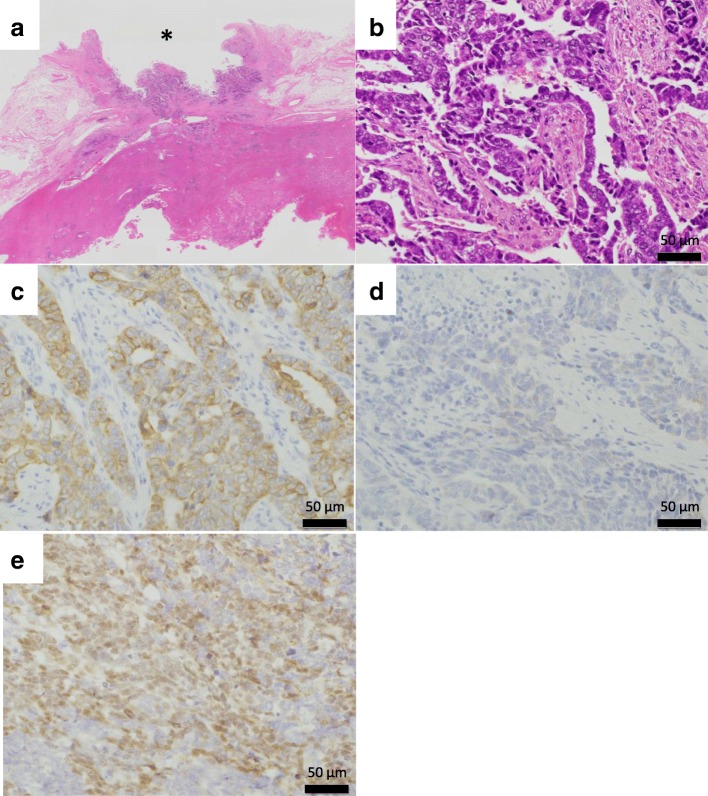
Pathological findings. **a** Hematoxylin-eosin (HE) staining indicated tumor cells were localized in the bile duct and no infiltration into the hepatic parenchyma was observed. *: bile duct lumen. **b** Atypical proliferation of cells with enlarged nuclei and exhibiting irregular ductal structures. Immunohistochemical staining revealed that the tumor cells were positive for CK-7 (**c**) and WT-1 (**e**), and negative for CK-20 (**d**)

Gross description indicated that the tumor was at the bifurcation of the common hepatic duct (Fig. 5). Histopathologically, tumor cells did not invade the liver parenchyma (Fig. 6a, b). The tumor was localized in the biliary wall and extended to the subserosal layer of the hepatoduodenal ligament, whereas direct exposure to the hepatoduodenal ligament was not detected. Dysplastic cells formed tubular structures and showed nuclear hyperchromasia. Psammoma bodies, which are small calcifications, were seen in the tumor. The histopathological features were similar to the pathology of the primary ovarian cancer. In the native biliary epithelium, no areas resembling dysplasia were seen. Peritoneal washing cytology suggested a serous adenocarcinoma. Immunohistochemical staining revealed that the tumor cells were positive for cytokeratin-7 (CK-7) and Wilms tumor-1 (WT-1) and negative for cytokeratin-20 (CK-20) and estrogen receptor (ER) (Fig. 6c–e). These findings suggested that the biliary tumor was derived from the ovarian cancer. Peritoneal metastases were scattered around the peritoneum, greater omentum, and diaphragm. The final diagnosis was an ovarian cancer-associated metastasis to the bile duct and recurrence of lymph node and peritoneal metastasis of ovarian cancer.

Her postoperative course was unremarkable. According to the chemotherapy sensitivity test, she received chemotherapy with paclitaxel against the residual peritoneal metastases for 7 months and achieved a clinical complete response. However, progression of the peritoneal dissemination and lymph node metastasis was confirmed 28 months after the operation.

## Discussion

Ovarian cancer spreads along the peritoneum or metastasizes through the blood or lymphatic stream. According to the large autopsy studies in ovarian cancer patients, common sites of metastases include the peritoneum, abdominal lymph nodes, bowel, liver, and lung, whereas the bile duct was not recognized as a metastatic site [6] [3]. The bile duct is suggested as an extremely rare site of ovarian cancer-associated metastases, as there are no previously reported cases in the literature.

Although rare, several cases of metastatic malignant tumor involving the bile duct have been reported. Intrabiliary growth of metastatic carcinoma was first reported in 1946 with symptomatic jaundice, biliary dilatation, and cirrhosis on autopsy [7]. Reported metastases to the bile duct include primary cancers of the colon, lung, breast, gallbladder, testicle, or pancreas [8]. The predominant primary lesions were colorectal carcinoma and the incidence of intrabiliary metastasis was reported as 3.6% in metastatic colorectal cancer [9]. Intrabiliary metastasis is extremely rare in non-colorectal tumors and its incidence was reported at 0.7% [9].

Several mechanisms for the intrabiliary growth of metastatic tumors have been suggested. Metastasis to the liver parenchyma through intrabiliary growth is suggested in colorectal cancer. Kubo et al. reported that macroscopic intrabiliary extension was observed in 10.6% of colorectal liver metastases. They also reported that in 3.7% of the cases, tumor formation was not observed in the liver parenchyma but only in the bile duct. As another mechanism, the peribiliary capillary plexus, which connects the portal vein or hepatic arteries, may help cancer cells metastasize to the bile duct via the blood stream [10]. In colorectal cancer, mucosal colonization in the biliary epithelium was commonly observed in intrabiliary metastasis [9]. Intraductal growth exhibits intraluminal expansion without dysplastic change in mucinous epithelium [11]. Further, cholangiocarcinoma spreads along epithelial surfaces with dysplastic changes around the tumor, which are well known as precursors of cholangiocarcinoma.

Imaging findings of intrabiliary metastasis mimic primary cholangiocarcinoma, and high-attenuation intraductal masses are regarded to be indistinguishable from the primary biliary malignancy [12] [13] [14]. When an intrabiliary tumor is found in patients with other malignant disease, diagnostic confusion with primary cholangiocarcinoma can occur. In this case, IDUS and CT showed an intraductal mass located at the hepatic hilum. Peritoneal dissemination and lymph node metastasis were not observed around the common bile duct. Tumor biopsy and brush cytology were suggested to be of benefit for a definite diagnosis of metastatic carcinoma [11]. However, in this case, preoperative endoscopic biopsy of the tumor showed atypical cells and did not provide a definite histopathological diagnosis. As cytologic or systemic diagnosis obtained during endoscopic retrograde cholangiopancreatography (ERCP) has low sensitivity, it is difficult to diagnose malignant etiologies of biliary strictures [15].

In this case, we performed a right hepatectomy with caudate lobectomy and extrahepatic bile duct resection, which is relatively invasive and can cause life-threatening complications. When she was diagnosed with a hepato-hilar bile duct tumor, recurrent peritoneal metastasis of ovarian cancer was also detected. The optimal treatment for metastatic ovarian cancer of the bile duct would be chemotherapy. However, in this case, we could not obtain a definite histopathological diagnosis preoperatively. In general, both paclitaxel and carboplatin are not effective in cholangiocarcinoma. There was concern that the tumor was a primary cholangiocarcinoma and these anticancer drugs would not be effective, resulting in a loss of opportunity for curative treatment. In this case, because infiltration to the right hepatic artery was suspected, the preoperative staging of the hepato-hilar tumor was estimated as stage IIIA by International Union Against Cancer (UICC) staging for perihilar cholangiocarcinoma. The overall 5-year survival rate after surgery in stage IIIA hilar cholangiocarcinoma is reported to be approximately 45% [16], whereas the 5-year survival rate of advanced ovarian cancer (FIGO Stage III/IV) is reported to be approximately 30% [17]. Surgical resection for cholangiocarcinoma was therefore selected as the optimal treatment.

In the resected specimen, the tumor was mainly located at the bifurcation of the common hepatic duct, and invasion to the liver parenchyma was not observed microscopically. Immunohistochemical staining was positive for CK-7 and WT-1, and negative for CK-20, suggesting that the tumor was not bile duct cancer but metastasis from ovarian serous adenocarcinoma [18]. The tumor extended to the subserosal layer of the hepatoduodenal ligament. However, direct exposure to the hepatoduodenal ligament was not detected histopathologically. Although a microscopic peritoneal metastasis was found at the hepatoduodenal ligament, this small tumor did not invade the bile duct and was completely separate from the tumor at the hepatic hilum. Finally, the patient was diagnosed with intraductal metastasis from ovarian cancer.

In conclusion, this is a rare case of ovarian cancer metastasis mimicking hilar cholangiocarcinoma at the bifurcation of the common hepatic duct. Whereas it is difficult to distinguish primary bile duct carcinoma from other metastatic disease, a correct diagnosis may be possible by considering the possibility of metastatic ovarian cancer and comprehensive evaluation of the medical history and the histological features.

## Abbreviations

CK-20: Cytokeratin-20
CK-7: Cytokeratin-7
CT: Computed tomography
ER: Estrogen receptor
ERC: Endoscopic retrograde cholangiography
FDG-PET: Fluorodeoxyglucose-positron emission tomography
FIGO: International federation of gynecology and obstetrics
IDUS: Intraductal ultrasonography
PTPE: Percutaneous transhepatic portal embolization
SUV: Standardized uptake value
WT-1: Wilms tumor-1

## References

[1] Jayson GC, Kohn EC, Kitchener HC, Ledermann JA (2014). Ovarian cancer. Lancet..

[2] Luvero D, Milani A, Ledermann JA (2014). Treatment options in recurrent ovarian cancer: latest evidence and clinical potential. Ther Adv Med Oncol.

[3] Guth U, Huang DJ, Bauer G, Stieger M, Wight E, Singer G (2007). Metastatic patterns at autopsy in patients with ovarian carcinoma. Cancer..

[4] Rosenblatt M, Zafaranloo S, Tancer ML (1989). Carcinoma of the ovary presenting as obstructive jaundice. Gynecol Oncol.

[5] Gupta A, Noba AL, Gupta S, Arora VK, Rathi V, Kumar S (2012). Papillary cystadenocarcinoma of ovary presenting as obstructive jaundice: a rare presentation. Oman Med J.

[6] Rose PG, Piver MS, Tsukada Y, Lau TS (1989). Metastatic patterns in histologic variants of ovarian cancer. An autopsy study. Cancer.

[7] Herbut PA, Watson JS (1946). Metastatic cancer of the extrahepatic bile ducts producing jaundice. Am J Clin Pathol.

[8] Menias CO, Surabhi VR, Prasad SR, Wang HL, Narra VR, Chintapalli KN (2008). Mimics of cholangiocarcinoma: spectrum of disease. Radiographics.

[9] Estrella JS, Othman ML, Taggart MW, Hamilton SR, Curley SA, Rashid A (2013). Intrabiliary growth of liver metastases: clinicopathologic features, prevalence, and outcome. Am J Surg Pathol.

[10] Ghittoni G, Caturelli E, Viera FT (2010). Intrabile duct metastasis from colonic adenocarcinoma without liver parenchyma involvement: contrast enhanced ultrasonography detection. Abdom Imaging.

[11] Riopel MA, Klimstra DS, Godellas CV, Blumgart LH, Westra WH (1997). Intrabiliary growth of metastatic colonic adenocarcinoma: a pattern of intrahepatic spread easily confused with primary neoplasia of the biliary tract. Am J Surg Pathol.

[12] Wenzel DJ, Gaede JT, Wenzel LR (2003). Intrabiliary colonic metastasis mimicking primary biliary neoplasia. Am J Roentgenol.

[13] Okano K, Yamamoto J, Okabayashi T, Sugawara Y, Shimada K, Kosuge T (2002). CT imaging of intrabiliary growth of colorectal liver metastases: a comparison of pathological findings of resected specimens. Br J Radiol.

[14] Moon SG, Han JK, Kim TK, Kim AY, Kim TJ, Choi BI (2003). Biliary obstruction in metastatic disease: thin-section helical CT findings. Abdom Imaging.

[15] Navaneethan U, Njei B, Lourdusamy V, Konjeti R, Vargo JJ, Parsi MA (2015). Comparative effectiveness of biliary brush cytology and intraductal biopsy for detection of malignant biliary strictures: a systematic review and meta-analysis. Gastrointest Endosc.

[16] Ebata T, Ercolani G, Alvaro D, Ribero D, Di Tommaso L, Valle JW (2016). Current status on cholangiocarcinoma and gallbladder cancer. Liver Cancer.

[17] Matz M, Coleman MP, Carreira H, Salmerón D, Chirlaque MD, Allemani C (2017). CONCORD working group. Worldwide comparison of ovarian cancer survival: histological group and stage at diagnosis (CONCORD-2). Gynecol Oncol.

[18] Bahrami A, Truong LD, Ro JY (2008). Undifferentiated tumor: true identity by immunohistochemistry. Arch Pathol Lab Med.

